# IN MEMORIAM Christopher Noel Williams December 25, 1935 to December 18, 2019

**DOI:** 10.1093/jcag/gwaa013

**Published:** 2020-04-09

**Authors:** Sander Veldhuyzen van Zanten

**Affiliations:** Division of Gastroenterology, Department of Medicine, University of Alberta, Edmonton, Alberta, Canada

Doctor Noel Williams passed away unexpectedly in his sleep on December 18, 2019 at 83 years of age. His given names reflect the fact that he was born on December 25.

**Figure:**
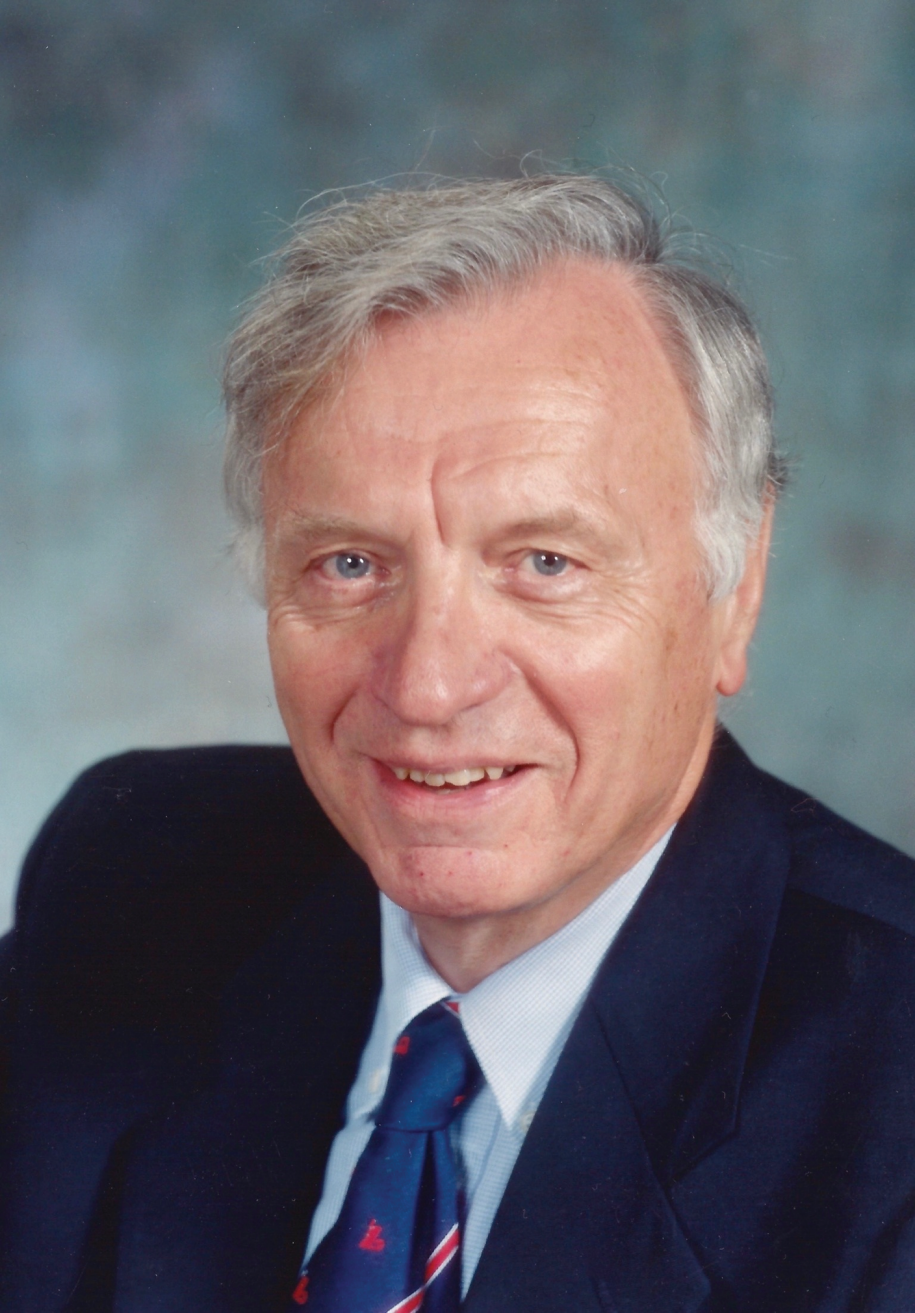


Noel has held important functions within the Canadian Association of Gastroenterology (CAG), and contributed significantly to the success of our organization. He was the CAG President from 1986 to 1987.

In 1992, he both received a research excellence award, and was the CAG Visiting Professor for excellence in research. In addition, he has held numerous other administrative functions including help with many aspects of CAG’s archives, helping to ensure the history of our organization’s achievements is preserved.

Importantly, together with Professor Alan Thomson, he was the founding father of the Canadian Journal of Gastroenterology (CJG), which first started publishing in 1987. Together they were the editors until 2001. Under their excellent stewardship, the journal flourished and became a key forum for academic publications from across Canada and beyond. Appropriately after their retirements they were elected as editors emeritus. An annual reward for best manuscript has been named in honour of both of them. This award has continued after the CJG morphed into the new Journal of the Canadian Association of Gastroenterology.

Noel was born in York, Yorkshire, UK, and moved to Canada in 1965. He did his initial medical training in Leeds. He worked for almost 2 years in Newfoundland before he entered residency training in Internal Medicine at Dalhousie University. After his Medicine training, he spent 2 years in Philadelphia, where he specialized in Gastroenterology. He started his first staff position at Dalhousie University in 1972.

Over the years, I personally have come to know Noel well. He was the Director of the Division of Gastroenterology at Dalhousie University in Halifax, when he recruited me in 1990. For 17 years from 1981 to 1998, Noel was an excellent Divisional Director. He provided always excellent advice to me as a new, young staff member both in clinical care, but also in research. He was a member of my PhD committee on the topic of *Helicobacter pylori* in 1992. He retired from Dalhousie University in 2001, at a time that retirement was mandatory. He certainly wanted to continue in Gastroenterology, and became a member of the Division of Gastroenterology at the University of Alberta in Edmonton in 2002. For me, it was surprising sequence of events that I saw him retire in Halifax, and four years later when I moved in 2006 to Edmonton to become the Head of the GI Division there, he again was a member of my Division. As before, his wise council and willingness to support all GI colleagues made him once again a very valuable member of our Division. He finally retired in 2010 at the age of 75.

Noel definitely also was a researcher at heart and his two passions were bile acids and liver disease and, separately, management of inflammatory bowel disease. At Dalhousie University, I remember his research office in the Annex building of the Faculty of Medicine well, which was stacked with papers in every conceivable space. There I had many memorable conversations with him. Apart from success in publishing in bile acids, he was a co-author on the first landmark paper on the use of cyclosporine as rescue medication for patients with severe Crohn’s disease, published in the New England Journal of Medicine in 1998.

Noel was married to the love of his life, Beryl, who he married in 1960, and by whom he is survived. They together raised a wonderful family of four children. Surely spending time with his kids and grandkids was one of Noel’s ongoing pleasures in life and he always talked about it. He loved the stock market and he also had an interest in cooking. Both in Halifax and Edmonton he and Beryl were members of a gourmet cooking club.

Noel will be fondly remembered as a wonderful giving person interested in people, and we thank him for all his contributions including the ones for CAG.

